# Experience with Clinically Diagnosed Down Syndrome Children Admitted with Diarrhea in an Urban Hospital in Bangladesh

**DOI:** 10.1155/2015/979404

**Published:** 2015-05-07

**Authors:** Rina Das, Anupam Sarker, Haimanti Saha, Abu Sadat Mohammad Sayeem Bin Shahid, K. M. Shahunja, Mohammod Jobayer Chisti

**Affiliations:** ^1^Dhaka Hospital, International Centre for Diarrhoeal Disease Research, Bangladesh (icddr, b), Mohakhali, Dhaka 1212, Bangladesh; ^2^National Institute of Preventive and Social Medicine (NIPSOM), Mohakhali, Dhaka 1212, Bangladesh; ^3^Centre for Communicable Diseases, icddr, b, Mohakhali, Dhaka 1212, Bangladesh; ^4^Centre for Nutrition & Food Security (CNFS), icddr, b, Mohakhali, Dhaka 1212, Bangladesh

## Abstract

There is lack of information in the medical literature on clinically diagnosed Down syndrome children presenting with diarrhea. Our aim was to describe our experience with Down syndrome patients admitted with diarrhea by evaluating the factors associated with Down syndrome presenting with diarrheal illness. In this retrospective chart analysis, we enrolled all the diarrheal children aged 0–59 months admitted to the Dhaka Hospital of the International Centre for Diarrheal Disease Research, Bangladesh (icddr, b), from March 2011 to February 2013. Down syndrome children with diarrhea constituted cases and randomly selected threefold diarrheal children without Down syndrome constituted controls. Among 8422 enrolled children 32 and 96 were the cases and the controls, respectively. Median age (months) of the cases and the controls was comparable (7.6 (4.0, 15.0) versus 9.0 (5.0, 16.8); *p* = 0.496). The cases more often presented with severe acute malnutrition, developmental delay, congenital heart disease, hypothyroidism, sepsis, hypocalcemia, developed hospital acquired infection (HAI) during hospitalization, and required prolonged stay at hospital compared to the controls (for all *p* < 0.05). Thus, diarrheal children with clinically diagnosed Down syndrome should be investigated for these simple clinical parameters for their prompt management that may prevent HAI and prolonged hospital stay.

## 1. Introduction

Down syndrome is popularly known as trisomy 21, because it is a genetic disorder caused by a presence of all or part of a third copy of chromosome 21, Patterson [1]. Globally, as of 2010, Down syndrome occurs in about 1 per 1000 live births [2] and results in about 17,000 deaths [3]. Most of the hospitals in Bangladesh do not have any facilities to confirm the diagnosis of Down syndrome using genetic sequencing. Thus, we often rely on clinical diagnosis of Down syndrome. Children with Down syndrome are more likely to have multiple health problems than the general population. Some of these problems are associated with failure to thrive leading to life threatening serious illness [3]. There is an increased incidence of gastrointestinal problems among people with Down syndrome [3]. It is therefore important to be aware of conditions involved and of their presenting symptoms and management. Although diarrhea may be a common comorbidity in Down syndrome children under 5 years, little is known about such population. Like other hospitals of Bangladesh and in other developing countries, the case-load of clinically diagnosed Down syndrome children having diarrhea is not uncommon at the Dhaka Hospital of the International Centre for Diarrhoeal Disease Research, Bangladesh (icddr, b), the only international organization that works on diarrhea disease research. However, there is no published data on the factors associated with clinically diagnosed Down syndrome children having diarrhea. Identification of factors associated with clinically diagnosed Down syndrome and diarrhea in such population may help to reduce morbidity and deaths in resource poor settings. Thus, the aim of our study was to describe our experience with Down syndrome patients admitted with diarrhea by identifying the factors associated with clinically diagnosed Down syndrome in diarrheal children.

## 2. Materials and Methods 

### 2.1. Ethics Statement

In this chart review, data were analyzed anonymously; thus, no parental or ethical consent was required.

### 2.2. Study Design

This was a retrospective chart review that was conducted at the Dhaka Hospital of icddr, b using electronic database of the hospital (SHEBA). We used an unmatched case control design and enrolled 8422 diarrheal children of both sexes, aged 0–59 months, who were admitted to the Intensive Care Unit (ICU), High Dependency Unit (HDU), or Longer Stay Ward (LSW) of the hospital from March 2011 to February 2013. Children with clinically diagnosed Down syndrome presenting with diarrhea constituted the cases and randomly selected diarrheal children without Down syndrome constituted controls. Controls were randomly selected by computer randomization using SPSS (version 17.0; SPSS Inc., Chicago) from a computerized data source of icddr, b. We used 1 : 3 unmatched case control ratio to increase the statistical power of our analyses. We defined Down syndrome clinically if a child had at least seven of the following clinical features: mental impairment, stunted growth, low muscle tone, depressed nasal bridge, slanted eyes, large protruding tongue, abnormal outer ears, wide gap between first and second toes, shortened head and neck, Simian crease, and high arch palate [4]. Diarrhea was defined if a child had three or more abnormally loose or watery stools per 24 hours, and status of dehydration was defined by “Dhaka Methods” of assessment of dehydration that is almost similar to WHO method and approved by WHO [5].

### 2.3. Study Site

The description of the study site (Dhaka Hospital of icddr, b) has been described in our previously published article [6].

### 2.4. Patient Management

Management of the diarrheal children with Down syndrome was done symptomatically; that is, management of pneumonia, sepsis, severe cholera, dysentery, severe malnutrition, and other bacterial infections was done following the hospital's guidelines [7]. The babies with Down syndrome were referred to Shishu Bikash Kendro of Dhaka Shishu Hospital or Bangabandhu Sheikh Mujib Medical University Hospital for further management and developmental rehabilitation.

### 2.5. Measurements

Case report forms (CRF) were developed and finalized for data acquisition. Characteristics analyzed included sociodemographic (age, gender, height and weight, type of residence, source of drinking water, and formula feeding), clinical signs (dehydration and temperature), clinical diagnosis (sepsis, pneumonia, chronic lung disease, pulmonary TB, severe acute malnutrition, congenital heart diseases (CHD), motor developmental delay within 0–59 months (neck control, sitting, standing with or without support, and walking) [8], hypothyroidism, hospital acquired infection (HAI) [9, 10]), and outcome during discharge. Sepsis was defined as presence or presumed presence of infection with hypothermia (≤35.0°C) or hyperthermia (≥38.5°C), tachycardia, tachypnea, and abnormal WBC count (>11 × 10^9^/L or <4 × 10^9^/L or band and neutrophil ratio ≥0.10) [11, 12]. Additionally, there may have been altered organ function such as altered mental status and bounding pulse in absence of clinical dehydration or after correction of dehydration. Pneumonia was initially diagnosed clinically following the World Health Organization recommended classification of pneumonia [13] and was confirmed with radiologic evidence of consolidation or patchy opacities [14]. Chronic lung disease was defined if there was moist cough for more than 4 weeks with recurrent chest infections, exertional dyspnea, and growth failure with or without the presence of clubbing, chest wall deformity, adventitious sounds on chest auscultation, and/or hyperinflation [15]. Severe acute malnutrition was defined as severe wasting (*z* score for weight for height/length, <−3 of the median of the WHO anthropometry), severe underweight (*z* score for weight for age, <−3 of the median of the WHO anthropometry), or nutritional edema. Pulmonary tuberculosis was diagnosed with supportive evidence such as positive tuberculin skin test or a positive contact history with a sputum positive tuberculosis patient plus if there was no improvement of signs of pneumonia or status of severe malnutrition following standard treatment plus even without microbiological confirmation of tuberculosis follow-up improvement with antitubercular therapy [6]. CHD was clinically diagnosed with auscultatory murmur heard by using stethoscope over cardiac area of the chest and was confirmed by echocardiography. Common CHD were atrioventricular septal defect or ventricular septal defect, mitral valve problems, tetralogy of Fallot, and patent ductus arteriosus [16]. Hypothyroidism was initially diagnosed clinically with any five of the following: history of feeding difficulties, failure to thrive, history of prolonged jaundice after birth, history of constipation, floppiness, large fontanelles, macroglossia, and cold mottled skin in the extremities and the diagnosis was confirmed by measuring TSH or thyroxine level in blood [17–19]. The identification of blood isolates and the susceptibility testing were done with the routine methods at the microbiological laboratory. Blood culture and serum electrolyte were processed in the Dhaka Hospital, icddr, b [20].

### 2.6. Analysis

All data were entered into SPSS for Windows (version 17.0; SPSS Inc., Chicago) and Epi-Info (version 6.0, USD, Stone Mountain, GA). Differences in proportions were compared by the Chi-square test. In normally distributed data differences of means were compared by Student's *t*-test and Mann-Whitney test was used for comparison of data that were not normally distributed. A probability of less than 0.05 was considered statistically significant. Strength of association was determined by calculating odds ratio (OR) and their 95% confidence intervals (CIs).

## 3. Results

There were 32 cases and 96 controls. The cases more often presented with developmental delay, congenital heart disease, hypothyroidism, severe acute malnutrition (SAM), sepsis, and lower serum calcium level compared to the controls. During hospitalization the cases more often developed HAI and required prolonged stay at hospital compared to the controls. All other variables among the cases and the controls shown in Table 1 were comparable.

## 4. Discussion

This study, though weakened by limited sample size, was able to describe our experience with Down syndrome patients admitted with diarrhea by identifying different associated factors for clinically diagnosed Down syndrome in diarrheal patients aged 0–59 months. Our observation of association of severe acute malnutrition, developmental delay, congenital heart disease, sepsis, hypothyroidism, and hypocalcemia on admission and prolonged hospital stay and HAI during hospitalization with Down syndrome in diarrheal patients is not surprising but needs to be reported for the better clinical management of such children.

Down syndrome is often associated with failure to thrive [21]. In our study, developmental delay was found to be strongly associated with Down syndrome in diarrheal children and the finding is consistent with previous study conducted in children without diarrhea [21]. Although there is a lack of evidence on the relationship between diarrhea and CHD, in this study, we have found strong association between CHD and Down syndrome in diarrhea patients. In our study we found 53% babies had CHD among Down syndrome and the observation is consistent with previous data which showed that the prevalence of CHD in children with Down syndrome involving nondiarrheal children was 40–50% [22, 23]. Thus, developmental delay, failure to thrive, and CHD might have an impact in contributing to SAM.

Our observation of association of sepsis and other infections (such as diarrhea and pneumonia) with Down syndrome in diarrheal children compared to those without Down syndrome is also understandable. This is probably due to the fact that children with Down syndrome in developing countries invariably present with SAM [24], that is, associated with immunosuppression, and often become prone to severe form of infection such as pneumonia, diarrhea, and sepsis [25, 26].

Our observation of the association of hypothyroidism with the diarrheal children having Down syndrome is also understandable. In our study we found 9.3% babies had thyroid dysfunction among Down syndrome whereas the prevalence of thyroid disease with Down syndrome in previous studies involving nondiarrheal children was 4–18% [17–19, 23, 27–29]. Down syndrome was also found to be significantly associated with hypocalcemia. Hypocalcemia used to occur mostly in children suffering from diarrhea and/or SAM [30] as in our study.

Although no published data were found on the association of HAI and prolonged hospitalization with Down syndrome in diarrheal children, the observation is quite understandable. In our study, diarrheal children with Down syndrome were observed to have had association with severe acute malnutrition, developmental delay, sepsis and various infections. Due to management of their multiple ailments, these children required to stay in the hospital for longer period which is strongly associated with HAI [31, 32].

The limitation of the study is the lack of genetic sequencing in diagnosing the children with Down syndrome. Another limitation is the small sample that might have an impact of the lesser association of other factors.

## 5. Conclusion 

Our data of Down syndrome in diarrheal children and the published data in nondiarrheal children suggest that the presentations of Down syndrome are almost similar in diarrheal and nondiarrheal children. Down syndrome in diarrheal children was found to have an association with severe acute malnutrition, congenital heart disease, hypothyroidism, developmental delay, sepsis, and hypocalcemia on admission. These children often required prolonged hospitalization and were prone to develop hospital acquired infection. Thus, clinicians may look for these simple clinical parameters in clinically diagnosed Down syndrome children with diarrhea for their prompt management that may prevent prolonged hospitalization as well as hospital acquired infection.

## Figures and Tables

**Table 1:** Characteristics of the cases and the controls admitted to the Intensive Care Unit/High Dependency Unit/Longer Stay Unit of the Dhaka Hospital of icddr, b.

Characteristic	Cases	Controls	OR (95% CI)	*p* value
	(*n* = 32)	(*n* = 96)	(unadjusted)	
Male	19 (59.4)	53 (55.2)	1.2 (0.49–2.9)	0.840
Age (months) (median, IQR) (range)	7.6 (4.0, 15.0)	9 (5, 16.8)	—	0.496
Temperature (Celsius) mean ± (SD)	37.3 ± 0.9	37.6 ± 1.07	—	0.151
Low socioeconomic status	20 (62.5)	61 (64.2)	0.9 (0.4–2.3)	0.960
Formula feeding	2 (2.1)	2 (1.6)	—	0.440
Developmental delay	17 (53.1)	3 (3.1)	64.5 (13.5–358.5)	<0.001
SAM	27 (84.4)	47 (49.5)	5.5 (1.8–17.9)	0.001
Dehydrating diarrhea	4 (12.5)	12 (12.5)	1.0 (0.27–0.43)	1.00
Pneumonia	22 (68)	43 (44.8)	2.7 (1.1–6.9)	0.320
Chronic lung disease	1 (3.1)	1 (1.0)	3.1 (0.0–116.2)	0.438
History of repeated RTI	1 (3.1)	3 (3.1)	1.0 (0.1–9.9)	1.00
Pulmonary TB	2 (6.3)	4 (4.2)	1.5 (0.2–10.5)	0.639
CHD	17 (53.1)	1 (1.0)	107.7 (13.2–2333.2)	<0.001
Hypothyroidism	3 (9.3)	0 (0.0)	—	0.006
Sepsis	3 (9.4)	0 (0.0)	—	0.010
HAI	5 (15.6)	2 (2.1)	8.7 (1.4–69.2)	0.010
Hypocalcemia (mmol/L)	4 (80.0)	2 (13.3)	26 (1.3–1187.8)	0.0139
Hypernatremia (mmol/L)	3 (16.7)	4 (12.9)	1.4 (0.2–8.6)	0.690
Total duration of hospital stay (median) IQR	6 (4.0, 10.0)	4 (2.0, 7.0)	—	0.009
Outcome (died)	1 (3.1)	1 (1.1)	0.3 (0.01–11.3)	0.410

Figures represent *n* (%) unless indicated otherwise.

OR = odds ratio; CI = confidence interval; IQR = interquartile range; SD = standard deviation.

Hypocalcemia = low serum calcium levels (ref. value = 2.12 to 2.62 mmol/L).

Hypernatremia = elevated sodium level in the blood (ref. value = 135–145 mmol/L).

HAI = hospital acquired infections; CHD = congenital heart disease; RTI = repeated respiratory tract infection; SAM = severe acute malnutrition.

## References

[1] Patterson D. (2009). Molecular genetic analysis of Down syndrome. *Human Genetics*.

[2] van Gameren-Oosterom H. B. M., Fekkes M., Buitendijk S. E., Mohangoo A. D., Bruil J., van Wouwe J. P. (2011). Development, problem behavior, and quality of life in a population based sample of eight-year-old children with down syndrome. *PLoS ONE*.

[3] Lozano R., Naghavi M., Foreman K. (2012). Global and regional mortality from 235 causes of death for 20 age groups in 1990 and 2010: a systematic analysis for the Global Burden of Disease Study 2010. *The Lancet*.

[4] Devlin L., Morrison P. J. (2004). Accuracy of the clinical diagnosis of Down syndrome. *The Ulster Medical Journal*.

[5] Alam N. H., Ashraf H. (2003). Treatment of infectious diarrhea in children. *Pediatric Drugs*.

[6] Chisti M. J., Graham S. M., Duke T. (2014). A prospective study of the prevalence of tuberculosis and bacteraemia in Bangladeshi children with severe malnutrition and pneumonia including an evaluation of Xpert MTB/RIF assay. *PLoS ONE*.

[7] Samadi A. R., Wahed M. A., Islam M. R., Ahmed S. M. (1983). Consequences of hyponatraemia and hypernatraemia in children with acute diarrhoea in Bangladesh. *British Medical Journal*.

[8] Keppler-Noreuil K. M., Welch J. L., Major H. J., Qiau Q., Jordan D. K. (2002). Atypical Down syndrome phenotype with severe developmental delay, hypertonia, and seizures in a child with translocation trisomy 21. *Developmental Medicine and Child Neurology*.

[9] Pien B. C., Sundaram P., Raoof N. (2010). The clinical and prognostic importance of positive blood cultures in adults. *The American Journal of Medicine*.

[10] Fagon J.-Y., Chastre J., Vuagnat A., Trouillet J.-L., Novara A., Gibert C. (1996). Nosocomial pneumonia and mortality among patients in intensive care units. *Journal of the American Medical Association*.

[11] Chisti M. J., Duke T., Robertson C. F. (2012). Clinical predictors and outcome of hypoxaemia among under-five diarrhoeal children with or without pneumonia in an urban hospital, Dhaka, Bangladesh. *Tropical Medicine and International Health*.

[12] Dellinger R. P., Levy M. M., Rhodes A. (2012). Surviving sepsis campaign: international guidelines for management of severe sepsis and septic shock. *Critical Care Medicine*.

[13] WHO (2013). *Pocket Book for Hospital Care of Children: Guidelines for the Management of Common Illness with Limited Resources*.

[14] Chisti M. J., Ahmed T., Faruque A. S. G., Abdus Salam M. (2010). Clinical and laboratory features of radiologic pneumonia in severely malnourished infants attending an urban diarrhea treatment center in Bangladesh. *Pediatric Infectious Disease Journal*.

[15] Chang A. B., Redding G. J., Everard M. L. (2008). Chronic wet cough: protracted bronchitis, chronic suppurative lung disease and bronchiectasis. *Pediatric Pulmonology*.

[16] Malt E. A., Dahl R. C., Haugsand T. M. (2013). Health and disease in adults with Down syndrome. *Tidsskrift for den Norske Laegeforening*.

[17] Shaw C. K., Thapalial A., Nanda S., Shaw P. (2006). Thyroid dysfunction in Down syndrome. *Kathmandu University Medical Journal*.

[18] Dayal D., Jain P., Panigrahi I. (2014). Thyroid dysfunction in Indian children with down syndrome. *Indian Pediatrics*.

[19] Chen M.-H., Chen S.-J., Su L.-Y., Yang W. (2007). Thyroid dysfunction in patients with Down syndrome. *Acta Paediatrica Taiwanica*.

[20] Shahunja K. M., Shahid A. S., Ashraf H. (2013). Predictors of death in under-five children with sepsis attending an urban diarrheal treatment centre in Bangladesh. *Food and Nutrition Sciences*.

[21] Hilhorst M. I., Brink M., Wauters E. A. K., Houwen R. H. J. (1993). Down syndrome and coeliac disease: five new cases with a review of the literature. *European Journal of Pediatrics*.

[22] Laursen H. B. (1976). Congenital heart disease in Down's syndrome. *British Heart Journal*.

[23] Bull M. J., Committee on G (2011). Health supervision for children with Down syndrome. *Pediatrics*.

[24] Bravo-Valenzuela N. J. M., Passarelli M. L. B., Coates M. V., Nascimento L. F. C. (2011). Weight and height recovery in children with down syndrome and congenital heart disease. *Revista Brasileira de Cirurgia Cardiovascular*.

[25] Suskind D., Murthy K. K., Suskind R. M., Suskind R. M., Suskind L. L. (1990). The malnourished child: an overview. *The Malnourished Child*.

[26] Golden M. H. N. (1998). Oedematous malnutrition. *British Medical Bulletin*.

[27] Moosa S., Segal D. G., Christianson A. L., Gregersen N. E. (2013). Thyroid dysfunction in a cohort of South African children with Down syndrome. *South African Medical Journal*.

[28] King K., O'Gorman C., Gallagher S. (2014). Thyroid dysfunction in children with Down syndrome: a literature review. *Irish Journal of Medical Science*.

[29] Cutler A. T., Benezra-Obeiter R., Brink S. J. (1986). Thyroid function in young children with Down syndrome. *The American Journal of Diseases of Children*.

[30] Chisti M. J., Salam M. A., Ashraf H. (2014). Prevalence, clinical predictors, and outcome of hypocalcaemia in severely-malnourished under-five children admitted to an urban hospital in Bangladesh: a case-control study. *Journal of Health, Population, and Nutrition*.

[31] Malhotra S., Sharma S., Hans C. (2014). Prevalence of hospital acquired infections in a tertiary care hospital in India. *International Journal of Medicine and Medical Sciences*.

[32] Ott E., Saathoff S., Graf K., Schwab F., Chaberny I. F. (2013). The prevalence of nosocomial and community acquired infections in a university hospital: an observational study. *Deutsches Ärzteblatt International*.

